# Continuous glucose monitoring identifies relationship between optimized glycemic control and post-discharge acute care facility needs

**DOI:** 10.1186/s13104-018-3656-3

**Published:** 2018-07-31

**Authors:** Scott M. Pappada, Karina Woodling, Mohammad Hamza Owais, Evan M. Zink, Layth Dahbour, Ravi S. Tripathi, Sadik A. Khuder, Thomas J. Papadimos

**Affiliations:** 1 Department of Anesthesiology, The University of Toledo College of Medicine and Life Sciences, 3000 Arlington Avenue, Toledo, OH 43614 USA; 2 Department of Bioengineering, The University of Toledo College of Engineering, 3000 Arlington Avenue, Toledo, OH 43614 USA; 3 Department of Anesthesiology, The Ohio State University Wexner Medical Center, 410 West 10th Avenue, Columbus, OH 43210 USA; 4 Department of Electrical Engineering and Computer Science, The University of Toledo College of Engineering, Toledo, OH 43606 USA; 5 Department of Medicine, The University of Toledo College of Medicine and Life Sciences, Toledo, OH 43614 USA; 6 Departments of Anesthesiology and Medical Microbiology and Immunology, The University of Toledo College of Medicine and Life Sciences, 3000 Arlington Avenue, Toledo, OH 43614 USA

**Keywords:** Glucose, Monitoring, Thoracic surgery, Extended care facilities, Algorithms, Clinical decision support systems

## Abstract

**Objective:**

Hyperglycemia is an independent risk factor in hospitalized patients for adverse outcomes, even if patients are not diabetic. We used continuous glucose monitoring to evaluate whether glycemic control (hyperglycemia) in the first 72 h after an intensive care admission was associated with the need for admission to a post discharge long-term medical facility.

**Results:**

We enrolled 59 coronary artery bypass grafting patients. Poor glycemic control was defined as greater than 33% of continuous glucose monitoring values < 70 and > 180 mg/dL (group 1); and then these patients were reevaluated with a less strict definition of poor glycemic control with greater than 25% of continuous glucose values < 70 and > 180 mg/dL (group 2). In group 1 4/10 (40.0%) whose glucose was not well controlled went to an extended care post discharge facility as opposed to 6/49 (12.2%) that were well controlled. In reevaluation as group 2, 5/14 (35.7%) whose glucose was not well controlled went to an extended care post discharge facility as opposed to 5/45 (11.1%) who were well controlled. Admission to a post discharge facility was increased in patients with poor glycemic control p = 0.045 and p = 0.042 for group 1 and group 2, and with odds ratios of 4.8 (95% CI 1.0–22.5) and 4.4 (95% CI 1.0–19.4), respectively.

## Introduction

There has been considerable study in regard to the importance of normoglycemia in the intensive care unit (ICU). Studies have shown a reduction in mortality, morbidity, and length of stay with adequate glycemic control in ICU patients [1, 2]. Hyperglycemia is frequent in hospitalized patients, and it is an independent risk factor for adverse outcomes, even if the patient is not diabetic [3]. There are subsequent studies that have tempered these claims and demonstrated that glycemic control that is too tight may be undesirable [4]. Our study examined whether the need for admission to a post discharge facility (PDF: a long-term acute care facility) from the ICU was associated with poor glucose control in the first 72 h after cardiothoracic surgery, as demonstrated through the use of continuous glucose monitoring (CGM). Regular use of CGM technology may allow better management of diabetic and non-diabetic patients with persistent hyperglycemia in the ICU, and thereby temper the need for post ICU care in a PDF. This was preliminary work done to support the development of a glucose clinical decision support system (CDSS) for the treatment of hyperglycemia in the intensive care unit (without identification of pre-existing life or life-styles). Such glucose data can be incorporated into algorithms that use additional information from the electronic medical record to create systems to assist in the care of critically ill patients.

## Main text

### Methods

This study was approved by the Ohio State University Wexner Medical Center Institutional Review Board. This prospective study enrolled 87 patients admitted to the Ross Heart Hospital ICU post-cardiothoracic surgery and from January 2014–June 2016; 59 were coronary artery bypass patients. Patients provided written informed consent. Inclusion criteria were a diagnosis of type 1 or type 2 diabetes mellitus, or no pre-existing diabetes diagnosis with initial glucose ≥ 150 mg/dL upon admission to the ICU. Exclusion criteria included pregnancy, prisoners, and those who did not have capacity to give consent. Each patient was subjected to CGM, which recorded interstitial glucose values every 5 min for the first 72 h of ICU admission. For this study, the Medtronic CGMS iPro 2 Recorder was used. This device recorded the interstitial glucose level every 5 min, and was applied to the abdomen of each subject. All information recorded in the patient’s electronic medical record was available for subsequent analysis. Normal glycemic control was defined as CGM values between 70 and 180 mg/dL per hospital guidelines. Here we confined our analyses to Ross Heart Hospital coronary artery bypass procedures 59/87 (67.8%) because of the small numbers of other surgical categories: 14 (16.7%) valve replacements, 10 (11.5%) ventricular assist devices, an infected graft excision (1.0%), a sternotomy (not valve or CABG) (1.0%), a femoral artery aneurysm repair (1.0%), and a lung transplant (1.0%).

Logistic regression along with Chi squared test were used to evaluate whether poor glycemic control in the first 72 h of ICU admission was indicative of PDF needs. Poor glycemic control was defined as patients with greater than 33% of CGM values outside of < 70 and > 180 mg/dL (group 1); and then these patients were reevaluated with a less strict definition of poor glycemic control with greater than 25% of CGM values outside of < 70 and > 180 mg/dL (group 2).

### Results

All patients in this study (n = 59 coronary artery bypass patients) completed the entire 72 h with a CGM device. This resulted in 864 glucose readings per patient and 50,976 glucose values overall. Distribution of study variables can be found in Table 1. None of the study variables differed significantly between the two groups so there was no need for an adjustment of variables.

**Table 1:** Distribution of study variables

	No PDF	+PDF	p-value
Sex			0.77
Male	34 (81%)	8 (19%)	
Female	15 (88%)	2 (12%)	
Age (years)	61.8 ± 9.0*	58.5 ± 7.2	0.23
BMI (kg/m^2^)	33.6 ± 7.9	35.3 ± 9.5	0.10
LOS ICU (days)	8.7 ± 8.5	15.0 ± 10.4	0.10
LOS hospital (days)	18.2 ± 13.5	15.0 ± 10.4	0.42

* Mean ± standard deviation; p = 0.05 is considered significant

*BMI* body mass index, *LOS ICU* length of stay in intensive care unit, *LOS hospital* length of stay in hospital

In group 1 4/10 (40.0%) whose glucose was not well controlled as defined went to a PDF as opposed to 6/49 (12.2%) who were well controlled. In reevaluation as group 2, 5/14 (35.7%) whose glucose was not well controlled as defined went to a PDF as opposed to 5/45 (11.1%) who were well controlled. The PDF requirement was significantly increased in patients with poor glycemic control, p = 0.045 and p = 0.042 for group 1 and group 2, respectively; and also in regard to odds ratios of 4.8 (95% CI 1.0–22.5) and 4.4 (95% CI 1.0–19.4), respectively (Table 2).

**Table 2:** Glucose values 33% and 25 outside of 70–180 ml/dL (n = 59)

	No PDF^c^	+ PDF	Totals
33% glucose out of range^a^			
Poor control	6	4	10
Good control	43	6	49
	49	10	59
25% glucose out of range^b^			
Poor control	9	5	14
Good control	40	5	45
	49	10	59

^a^p = 0.045; odds ratio = 4.8 (95% CI 1.0–22.5)

^b^p = 0.042; odds ratio = 4.4 (95% CI 1.0–19.4)

^c^PDF = post discharge facility

**Fig. 1:**
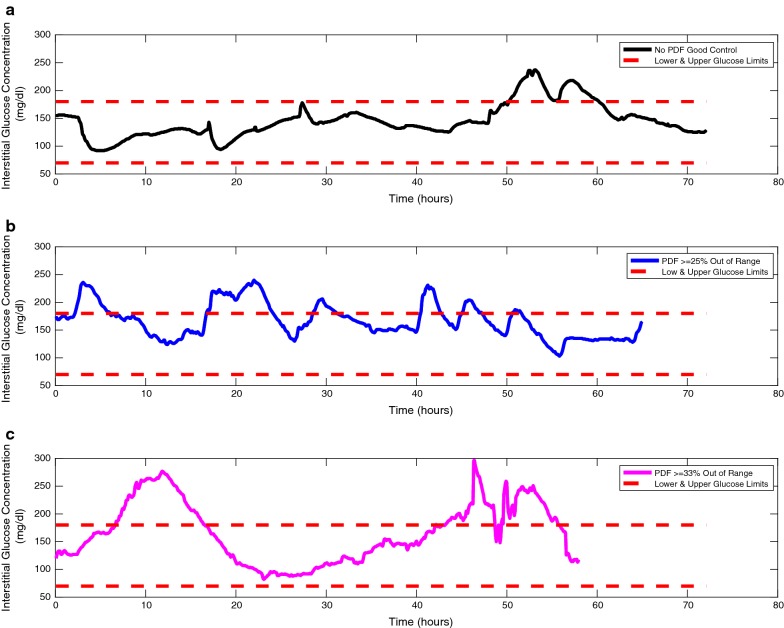
Good glucose control (**a**), and poor glucose control (**b**, **c**), > 25 and > 33% out of range

Figure 1 depicts a graph of three patients, one with good glucose control and two others with glucose > 25 and > 33% of values out of range. This figure provides a visual perspective of the importance of glycemic control and how continued and/or prolonged excursions beyond the control limits (dotted red lines) can be associated with, or be cautionary of, a particular ICU patient’s risk for transfer to a PDF from an ICU.

### Discussion

In this study we determined an association between hyperglycemia and the need for care at a PDF after a patient underwent a CABG procedure. We evaluated the patients using two different definitions of glycemic control, that of group 1 were stricter than group 2, and in both cases the requirement for a PDF was significantly increased. In the group 1 (> 33% of glucose values out of range) and group 2 (> 25% of values out of range) there was an association with an increased need for transfer to a PDF directly from the ICU. The degree of glycemic control in a CABG surgery ICU patient population may be a predictor of PDF care needs, and may contribute to increased costs, as well as morbidity and mortality. The authors understand that glycemic control is only one aspect of care among a host of factors that may contribute to extended care in a PDF. Nonetheless, our acquisition of 50,976 data points among the 59 patients provided a level of confidence to suggest that glucose control among our patients in the first 72 h of their ICU stay may be a noteworthy associated contributor, or marker, of a need for care in a PDF and indicated in both groups by P value and odds ratios. Additionally, Fig. 1 lends itself to allowing the reader to visualize the importance of excursions outside of the glucose ranges of 70–180 mg/dL.

Hyperglycemia is an independent risk factor for poor outcomes in those with and without diabetes, generally [3]. Many, but not all trials in regard to hyperglycemia show benefits of glucose control [3]. CGM may better assist health care providers in managing glycemic control in ICU patients and contribute to improved quality and safety. Maintaining normoglycemic control within this population, with or without diabetes, is of paramount importance [3, 5, 6]. Additionally, subcutaneous CGM is safe in critically ill patients in terms of hypoglycemia [6]. Boom et al. make the important point that algorithms in CGM play a critical role, and more work and research along these lines is quite important [6]; something our research team is pursuing. Additionally, Sleiman et al. studied the impact of hyperglycemia on older patients, and concluded that in those most ill, the need for controlling hyperglycemia is extremely important because of its association with an increased mortality at 45 days; and, in fact, may be more important to control and follow in those patients without diabetes than in those patients with diabetes [5].

Also, the fact that surgical site infections in the older patients are more frequent, deadlier, have higher costs, and are related to a decreased host response, make the optimization of perioperative glycemic control very important [7]. When studying the biological considerations of enhanced recovery after surgery programs (ERAS), it is evident the inflammatory stress response of surgery with the accompanying release of counter regulatory hormones, leads to insulin resistance, which if not addressed is a source of adverse outcomes in patients [8]. Recent direct evidence has identified a genetic pathway in which acute hyperglycemia can obliterate the cardioprotective effect of remote ischemic preconditioning through nitrative stresses and activation of the phosphorylation of the rapamycin pathway [9]. Additionally, inflammation of the central nervous system secondary to anesthesia, the surgical insult, and cardiopulmonary bypass may initiate rapid neurological and cognitive decline in patients [10], and this effect can be compounded by a lack of perioperative glycemic control [11–14]. In this study we used CGM in caring for our patients; its use in the critically ill is safe, economical, and can decrease nursing workload [6]. The use of CGM is supported by the 2016 Consensus Statement of the American Association of Clinical Endocrinologists and the American College of Endocrinology [15]. The Association and the College state that CGM improves the control of glucose, reduces costs in the care of diabetics, and will likely improve the outcomes of diabetics.

While glucose control is important, how tight or how liberal glucose levels should be is still being debated. While hypoglycemia in the intraoperative period for cardiothoracic surgery is associated with delirium in adults [13, 16], Zhang et al. reported that hyperglycemia was associated with new postoperative cognitive problems in adults after cardiothoracic surgery, and that new protocols regarding glycemic control should be pursued [12]. Also of interest is that better and tighter control of glucose may be of benefit in children who undergo complex congenital heart procedures [17].

This preliminary work was done in order to create a basis for development of a glucose CDSS for clinicians. Such a system will be based on algorithms using the electronic medical record and an artificial neural network. By incorporating an increased frequency of information collection through standard glucose monitoring and in the future CGM use, along with other electronic medical record data, clinicians may be able to better assist health care providers in optimizing glycemic control in the ICU setting. The data acquired in this study may be a contribution to defining the need for long-term acute care utilization in cardiothoracic surgery patients after ICU discharge, especially if incorporated into a CDSS. We encourage our colleagues with interest in glycemic control in the cardiothoracic surgery perioperative period to pursue monitoring and algorithm strategies in regard to continuous glucose monitoring and glucose CDSS in order to deliver better outcomes to our cardiothoracic surgery patients that also result in a cost-savings to society.

## Limitations

There are limitations to our study. Our work, while hoping to target an older population was confounded by the occasional younger person who was taken for a cardiothoracic procedure. The study was limited, intermittently, by the number and availability of CGM devices. Additionally, there may be differences in interstitial glucose levels measured by CGM when compared to those which are directly take from the blood at the point of care. Furthermore, while we have different surgery procedures, we cannot comment on pre-existing diseases, or life-styles. Also, this work was part of a broader study where we intend to report how well post cardiothoracic surgery patients blood glucose is controlled by point of care vs. CGM, and thereafter create a CDSS. Finally, we only documented the glucose levels for the first 72 h of the patients’ ICU stay, although the patient length of stays varied. However, we felt obligated to report what was observed in regard to how the lack of optimal glycemic control (hyperglycemia) was associated with the need for post ICU discharge to an extended care facility.
